# Processability of Ancient Wheats for Novel Value Chains and Agro-Food Biodiversity

**DOI:** 10.3390/foods15050929

**Published:** 2026-03-06

**Authors:** Francesca Nocente, Diana DeSantis, Marta Naso, Gaia Blandizzi, Margherita Modesti, Serena Ferri, Gabriele Chilosi, Laura Gazza

**Affiliations:** 1 CREA Research Centre for Engineering and Agro-Food Processing, Via Manziana 30, 00189 Rome, Italy; francesca.nocente@crea.gov.it (F.N.); marta.naso@crea.gov.it (M.N.);; 2 Department for Innovation in Biological, Agro-Food and Forest Systems (DIBAF), University of Tuscia, Via San Camillo de Lellis snc, 01100 Viterbo, Italy; desdiana@unitus.it (D.D.); margherita.modesti@unitus.it (M.M.); serenaferri@unitus.it (S.F.); chilosi@unitus.it (G.C.)

**Keywords:** *Triticum*, ancient wheat, pasta, bread, agri-food biodiversity

## Abstract

Modern wheat breeding has focused on maximizing yields under high-input systems. Although ancient wheat varieties generally show lower yields and no clear nutritional superiority, they are increasingly valued in organic and local food systems for their resilience, cultural identity, and suitability for artisanal processing. This study evaluated the physicochemical, rheological, and technological properties of stone-milled flours and *semolato* from ancient common, durum, and Khorasan wheat to develop artisanal bread and pasta. Ancient cultivars showed relatively high protein content, ranging from 10.9% to 15.9% (on a dry matter basis). Gluten quality was generally weak, with gluten index values below 30% in most cultivars and alveograph W values below 60 × 10^−4^ J, mainly in durum wheats. Among common wheat cultivars, Autonomia B and Rano Solina showed the best bread-making suitability and were selected to produce bread prototypes via the application of pre-gelatinization. Optimized fermentation and pre-gelatinization significantly improved the crumb structure, softness, and sensory acceptance. Pasta from durum cv. Senatore Cappelli and Khorasan showed good cooking and sensorial quality, with Khorasan receiving a better score for overall acceptability. This study demonstrates that appropriate processing strategies can successfully unlock the technological and sensory potential of ancient wheat varieties, supporting their use in short value chains and enhancing product differentiation.

## 1. Introduction

During the last century, wheat breeding has primarily focused on increasing yields. As a result, modern varieties are characterized by genetic uniformity and adaptation to conventional agriculture that relies on high energy inputs in terms of fertilizers, herbicides, insecticides, and fungicides [1], Moreover, modern varieties are often selected under favorable environmental conditions that do not adequately represent the diversity of local agroecological contexts.

The development of local and participatory agri-food value chains, from primary production to the transformation of raw materials into high-nutritional and high-added-value products, is increasingly recognized as a cornerstone of sustainable food systems [2,3]. Such approaches foster territorial development, enhance farmer engagement, and support socioeconomic resilience, while addressing the negative externalities of globalized food systems, including biodiversity loss and the cultural homogenization of taste [4]. Organic farming, particularly that based on diversified rotations that include legumes, plays a critical role in improving soil fertility, reducing synthetic inputs, and promoting agroecological sustainability [5,6]. These practices support key ecosystem services such as nitrogen fixation, carbon sequestration, and biodiversity conservation, which are essential for climate change mitigation and resilience [7]. At the same time, globalization and dietary standardization have accelerated the erosion of agro-food biodiversity, replacing complex ecosystems with monocultures and uniform food products [4,8]. Ancient wheat varieties are often perceived as nutritionally superior; however, current evidence indicates that they do not outperform modern cultivars in terms of nutritional composition or technological performance, and they generally exhibit lower yields [9,10,11,12]. Despite these limitations, ancient wheat varieties possess distinctive added value as niche products, primarily because they convey cultural heritage and authenticity—attributes that resonate strongly with consumers and producers within short, participatory supply chains [13]. The recent repositioning of cereals from commodity to specialty products has enhanced opportunities for market differentiation and narrative-based valorization, which are increasingly relevant in contemporary agri-food markets. Ancient wheat varieties exhibit notable yield stability across different environmental conditions, even though their overall yield is generally lower than those of modern, high-input need varieties. This intrinsic stability constitutes inherent agronomic resilience, making these varieties particularly well suited for sustainable, low-input farming systems [12,14]. Their adaptability to marginal environments further supports their integration into agroecological practices and climate-smart agriculture [15].

In the last decade, the cultivation of ancient wheat varieties has contributed to the development of local food micro-economies, enabling producers to differentiate their products and increase their added value through consumer-recognized attributes, such as “locally produced” labels. Moreover, these grains are mostly processed by small-scale local processors into semi- or wholemeal flours, rather than refined flours or semolina, as this approach allows for higher flour yields while enhancing the nutritional quality and sensory properties of the resulting products [16,17,18]. Indeed, wholewheat foods are increasingly recognized for their rich content and compositions of bioactive components, including dietary fiber, phenolic compounds, vitamins, and minerals. These constituents contribute to a wide range of health-promoting effects, such as improved glycemic control, enhanced gut microbiota diversity, and reduced risks of chronic diseases like cardiovascular disorders and type 2 diabetes [19]. However, the retention of the bran and germ in wholegrain foods negatively affects their shelf life and palatability, which represents a major technological challenge in wholegrain processability and consumer acceptance [20]. Moreover, plant-based diets that prioritize whole grains, along with legumes and nuts, tend to have a significantly lower environmental footprint, reducing greenhouse gas emissions, land use, and water consumption, compared to diets dominated by refined grains [21].

Within this scenario, the present study focused on the development of two traditional Italian foods—pasta and bread—that are an integral part of the Italian culinary heritage and constitute key components of the Mediterranean diet. Wholemeal pasta and bread, from Italian ancient wheat varieties of durum and common wheat, respectively, were evaluated for their technological and sensorial quality, with the aim of contributing to unlocking the potential of ancient cultivars by enhancing their valorization within high-added-value agri-food chains.

## 2. Materials and Methods

### 2.1. Flour Materials

Organic stone-milled *semolato* of *T. turgidum* ssp. *durum* cvs Senatore Cappelli and Timilia, *T. turgidum* ssp. *turanicum* (hereafter “Khorasan”), and stone-milled flours of *T. aestivum* L. cvs Andriolo, Autonomia B, Gentil Rosso, Maiorca, Rano Solina, and Verna were kindly supplied by the agricultural companies Fornovecchino (Montefiascone, Viterbo, Italy) and Umberto Di Pietro (Capena, Italy),partners of the research project.

### 2.2. Physicochemical Characterization

Protein content was determined according to the Dumas method [22], using Nx5.7 as the conversion factor. Ash content was determined according to the approved AACC method 08-01.01 [23]. Moisture content was measured using a thermobalance (Sartorius MA 40, Göttingen, Germany) at 120 °C shortly before the chemical analyses. The color of flour, *semolato*, and pasta samples was evaluated with a tristimulus colorimeter (Chroma Meter CR-400; Minolta, Osaka, Japan), using the CIELab color space coordinates L* (lightness), a* (red–green chromaticity), and b* (yellow–blue chromaticity) and the D65 illuminant. All analyses were performed in triplicate, and the data were expressed as dry matter (d.m.).

### 2.3. Technological and Rheological Characterization

Flours were analyzed via a Chopin alveograph (Chopin, Villeneuve La Garenne, France) according to the manufacturer’s instructions, as described in the standard AACC method 54-30.02 [24]. The alveograph analysis of *semolato* was performed using a protocol adapted for durum wheat, as described in [25]. The SDS sedimentation test was performed according to the AACC standard method 56-70.01 [24]. Gluten index determination was conducted with the Glutomatic 2200 (Perten Instruments, Segeltorp, Sweden), according to the AACC method 38-12 [24]. The farinographic test was conducted using a Brabender farinograph (Duisburg, Germany), following the ISO standard method [26]. The Perten 1500 system (Perten Instruments) was used for the determination of the falling number, according to the AACC standard method 56-81B [24]. All parameters were measured in triplicate.

### 2.4. Baking Trials and Bread Sensory Analysis

Two prototypes were developed to assess the baking performance of the selected flours (Rano Solina and Autonomia B) under controlled laboratory conditions. A planetary mixer and a ventilated oven were used for all trials, and proving was conducted in a temperature-controlled chamber at 30 °C. A mature sourdough starter was used as the leavening agent in all bread formulations. Sourdough was formed by fermenting a mixture of flour (Autonomia B and Rano Solina) and water at a ratio of 1:1, without the addition of an external starter culture. Every 24 h, the refreshing procedure was carried out by adding 100 g of fermented dough, 100 g of flour, and 100 mL of water.

For the first pan loaf prototype (Prototype A), prepared using Rano Solina flour, the optimized dough formulation included 280 g flour, 168 g water, 70 g sourdough starter, one teaspoon of salt, and 1.5 tablespoons of extra-virgin olive oil. Dough mixing followed the same procedure as above, with two manual folds applied during the first 10 min of rest. Dough development was monitored by recording the height/volume at different fermentation times (T0, 3.5, 4, 5, and 6 h), and acidity was assessed by pH measurement. The ideal fermentation time was 3 h 30 min, after which the dough was transferred to a loaf pan and baked at 180 °C for 43–45 min.

For the second pan loaf prototype (Prototype B), formulated with Autonomia B flour, a pre-gelatinization step applied to 10% of the total flour was included to address the compact structure observed in preliminary trials in order to improve dough hydration and structure. Specifically, 28 g of flour was mixed with water at a 1:3 ratio, and the mixture was heated at approximately 80 °C until complete starch gelatinization occurred. The resulting gel was then incorporated into half of the remaining flour, previously premixed with water and oil, and subjected to an initial low-speed mixing phase. After this pre-mixing step, the rest of the flour was gradually added until a homogeneous and workable dough was obtained, ready for shaping and baking. Fermentation was conducted for 5 h, and baking took place at 180 °C for approximately 45 min. The pre-gelatinized fraction was expected to improve dough hydration, gas retention, crumb softness, and overall loaf development.

Sensory analysis was performed exclusively on breads produced with flour from the Autonomia B wheat cultivar (Prototype B), as the pre-gelatinization step was applied only for this formulation, allowing a direct comparison between gelatinized and non-gelatinized products. Two samples were therefore evaluated: bread produced using a traditional process with 5 h of leavening and bread produced with the inclusion of a pre-gelatinization step for the dough.

The sensory analysis was conducted by a 12-member panel of experts selected based on their experience in food product evaluation, the regular consumption of bakery products, and the absence of gluten allergies. The panelists were trained in accordance with the standard method ISO 8586:2023 [27]. Assessments were carried out under controlled laboratory conditions, ensuring standardized lighting, temperatures, and humidity, according to the ISO 8589:2007 standard method [28]. A descriptive sensory analysis was carried out by the panel using a product characterization test based on quantitative intensity ratings of sensory descriptors, following the method ISO 13299:2016 [29], with the aim of identifying the most relevant attributes for discriminating between samples.

### 2.5. Pasta-Making Process

Artisanal pasta formulations from the stone-milled *semolato* of Khorasan wheat and durum wheat cv. Senatore Cappelli were produced by the local small-scale farmer–processor Fornovecchino (Viterbo, Italy). The appropriate consistency of the doughs for the extrusion process was achieved by hydrating the *semolato* to a level of 32% humidity. The artisanal pasta-making setup was equipped with a press (Dominioni Punto & Pasta P60, Como, Italy) with a capacity of up to 60 kg/h, which ended with a bronze-coated die (127 mm diameter) to produce a macaroni shape. Pasta samples were dried for 18 h in a dryer (La Parmigiana, Fidenza-, Italy), applying a low-temperature drying program (T max = 42 °C) and a linear decrease in the relative humidity in the drier chamber from 85% to 70% throughout the entire drying process. The final moisture content of dried spaghetti was 12.5%.

### 2.6. Pasta Cooking Quality and Sensory Analysis

Pasta cooking quality was determined by a cooking test, according to the AACC method [30]. Specifically, 100 g of dried pasta was cooked in 1 L of boiling tap water until the optimal cooking time (OCT) was reached, corresponding to the disappearance of the starchy central core [31]. Water absorption (WA) was calculated from the weight increase of pasta at the OCT and determined as WA = [(w − w0)/w0] × 100, where w and w0 are the weight of cooked and raw pasta, respectively.

Sensorial judgment was based on two textural parameters of cooked pasta: firmness and stickiness. Each parameter was evaluated using a score ranging from 10 to 100, assigned by a trained panel of five assessors [31] and expressed as the arithmetic mean of the five scores. The global sensorial judgment (GSJ) score was the arithmetic mean of the two textural components. For stickiness, ≤20 = very high, >20 and ≤40 = high, >40 and ≤60 = rare, >60 and ≤80 = almost absent, >80 and ≤100 = absent; for firmness, ≤20 = absent, >20 and ≤40 = rare, >40 and ≤60 = sufficient, >60 and ≤80 = good, >80 and ≤100 = very good. The global sensorial judgment score ranged from 10 to 100, where <55 = scarce, ≥55 and <65 = sufficient, ≥65 and <75 = good, ≥75 = very good.

### 2.7. Statistical Analysis

The results were presented as the mean ± standard deviation. A one-way analysis of variance was conducted using the MSTAT-C v.2.1. software (Michigan State University, East Lansing, MI, USA), followed by Duncan’s multiple range test for the post hoc comparison of the means, in order to evaluate the significance of differences (*p* ≤ 0.05) for each measured parameter. Sensorial bread descriptor data were processed using the XLStat Addinsoft 2020.1.1. (Bègles, France). For each descriptor, a two-way ANOVA model was applied, considering product and judge as fixed effects, in order to assess the significance of differences between samples and the variability associated with panelists. The statistical output provided model coefficients for each product–descriptor combination. The discriminating power of each descriptor was assessed using t-tests, identifying the descriptors contributing most to differentiation between samples with pre-gelatinization (AP) and those without pre-gelatinization (AC).

## 3. Results and Discussion

### 3.1. Physicochemical Characterization of Milling Products

Besides their nutritional value, wheat storage proteins are considered the most important technological aspect influencing bread and pasta processability, as they play a crucial role in obtaining a suitable dough. The mean protein content of the ancient common wheat varieties was 12.7 g/100 g, ranging from 10.9 to 15.9 g/100 g (Table 1), recorded in Autonomia B and Rano Solina, respectively. Among the *T. durum* varieties, the mean value was 11.5 g/100 g, and the highest protein content was observed in cv. Senatore Cappelli, followed by Timilia and Khorasan wheat (Table 1). It should be emphasized that these old varieties were grown under organic farming conditions; therefore, the relatively high protein content highlights their good performance in low-input agricultural systems. Ash content is an important parameter in the assessment of flour quality, from both a nutritional and technological point of view. It reflects the flour mineral amount and is mainly influenced by the milling process and the flour extraction rate. The ash content of the samples (Table 1) was evaluated in relation to the limits established by Italian law (DPR No. 187/2001), which defines maximum ash levels for wheat flours and semolina on a dry matter basis according to their degrees of refinement. In the flours of common ancient wheat varieties, the ash values ranged from 0.73 to 1.13 g/100 g. According to the commercial classification criteria established by Italian legislation, the flours could be classified as semi-wholemeal flours. Specifically, the flours obtained from cvs. Andriolo, Rano Solina, and Verna were classified as Type 2 (ash content maximum 0.95 g/100 g). Similarly, the Gentil Rosso and Maiorca samples were classified as Type 2 flours, as their ash content exceeded 0.95 g/100 g but did not reach the minimum threshold established for wholemeal flours (1.30 g/100 g). Cultivar Autonomia B was the only one classified as a Type 1 flour (ash content maximum = 0.80 g/100 g). As expected, the two *semolato* samples showed significantly higher ash content than the flours (Table 1), slightly exceeding the Italian legal limits (DPR No. 187/2001), reflecting both the milling process and the inherently higher endosperm ash levels of durum wheat [32]. Specifically, *semolato* is the product obtained from the milling and sieving of durum wheat after the extraction of semolina [33]. The color of flour and *semolato* is a key quality attribute, as it strongly influences the color of the final product, such as bread crumb and pasta, which in turn affects consumer perception and acceptance. Color characteristics are determined by the content and composition of pigments, particularly carotenoids, and by the flour extraction rate and flour particle size. Among the common wheat samples, Autonomia B showed the lowest 100-L* values—corresponding to a higher brightness index, associated with lower ash content (Table 1)—when compared to the other flour samples, which showed higher ash content accompanied by higher brownness. These results are consistent with previous findings [34] that suggested the contribution of mineral-rich outer kernel fractions to a dark color, since bran particles and other pigmented compounds negatively affect flour brightness. Differently from brownness, the flour yellow index (b*) was not directly affected by the ash content but was mainly influenced by the genotype (Table 1). Autonomia B and Rano Solina exhibited the lowest b* values, whereas no significant differences were detected among the other cultivars.

It is well known that the yellow index (b*) is particularly important for durum wheat’s commercial value, since regular consumers of semolina pasta favor products with a bright yellow color. Compared to common wheat flour, *semolato* from old durum wheat cultivars showed significantly higher values of both brownness (100-L*) and yellowness (b*) (Table 1).

This difference can be mostly attributed to the larger particle size of *semolato*, resulting from the coarser grinding of the inherently harder durum wheat endosperm, which affects color perception independently of the chemical composition, by altering light scattering and leading to lower L and higher b* values, even at comparable pigment concentrations [35]. Among the *semolato* samples, Khorasan wheat showed a higher b* value, which reflects the characteristic amber color of its grains. A higher brownness value (100-L*) was found in cv. Senatore Cappelli, likely as a consequence of its high ash content (Table 1).

### 3.2. Technological and Rheological Characterization

As shown in Table 2, all *T. turgidum* genotypes exhibited poor gluten quality, as indicated by gluten index (GI) values consistently below 10%, SDS sedimentation volumes lower than 30 mL, and alveograph W values below 60 J × 10^−4^, with the Timilia variety reaching values as low as 37 J × 10^−4^. Furthermore, as highlighted by the P/L ratio and the elasticity index (Ie), which was practically null, the doughs proved to be particularly tenacious, especially for the Cappelli and Khorasan varieties, with very limited extensibility. These findings suggest that these varieties are more suitable for producing low-leavened breads or products that do not require intensive processing, e.g., for pasta-making or biscuits. Among the common wheat varieties, only cv. Autonomia B exhibited an excellent gluten index (>80%), whereas cv. Rano Solina demonstrated medium gluten quality (30% < GI < 80%). The remaining varieties showed markedly poor gluten quality (GI < 30%), with cvs. Maiorca and Gentil Rosso displaying the lowest values (Table 2). All common wheat varieties examined fell within the first quality class (>40 mL) based on the SDS sedimentation test values. As indicated by the alveograph W value, the cultivar exhibiting the highest gluten strength was cv. Autonomia B, although it was consistently below 100 J × 10^−4^ (Table 2). For bread-making purposes, the P/L ratio should not exceed 1 to give a high loaf volume [36], with an optimum range of 0.4 to 0.8 [37,38]. The P/L ratio and elasticity index (Ie) values of the analyzed common wheat varieties were generally well balanced, suggesting good bread-making suitability, although Maiorca, Gentil Rosso, and Autonomia B showed reduced dough extensibility (Table 2). The farinograph test confirmed the low gluten strength previously assessed through the GI and alveographic parameters. Specifically, this analysis evaluates flour behavior during kneading. In this context, desirable characteristics for wheat flour include a long dough development time, a high water absorption capacity, elevated stability values, and a low degree of softening [37,38]. The water absorption values of the samples ranged from 52.3 to 62%, with cvs. Andriolo and Verna showing the lowest values, whereas Rano Solina, Maiorca, and Senatore Cappelli were characterized by higher water absorption capacities (Table 2), likely due to their higher protein content (Table 1). Indeed, water absorption is closely related to the amount of protein, besides the presence of damaged starch and non-starch carbohydrates and the flour particle size [39,40]. These results confirm the weak gluten structures of these varieties, which require less water to obtain doughs of the desired consistency [40]. The dough development time is influenced by the amount and quality of gluten in the flour, as well as by its water-binding capacity [41]. In this study, all ancient wheat varieties showed short development times (on average 2.2 min), associated with low stability (on average 2.2 min) and with a high degree of softening (Table 2).

Based on the results, the analyzed flours can be classified as weak flours, thus confirming the outcomes of the alveographic analysis. Nevertheless, Rano Solina proved to have better performance for bread production, mainly in terms of water absorption, the development time, and the degree of softening. Finally, the falling number (FN) values, an indicator of wheat flour’s baking quality in relation to amylase activity, were found to be very high (>450 s), mainly in *T. turgidum* varieties (Table 2). When the FN exceeds 300 s, it indicates low amylase activity in the flour, which may result in delayed fermentation and products characterized by a hard crust and reduced loaf volume. Among the wheat cultivars analyzed, only Autonomia B and Rano Solina proved to have good bread-making suitability.

### 3.3. Prototype A—Pan Bauletto (Rano Solina 100%)

**Figure 1:**
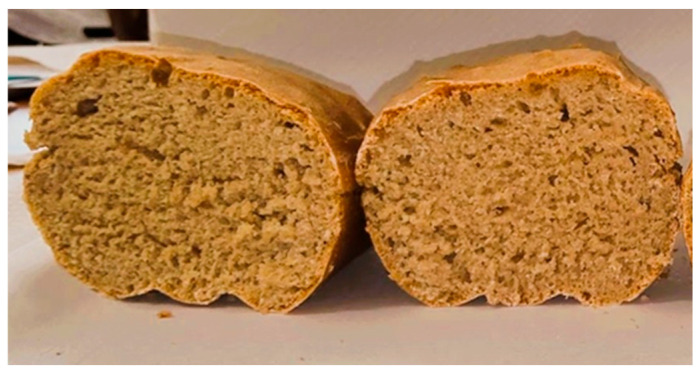
Cross-sectional view of bread samples produced with flour from the Rano Solina wheat cultivar.

The pan loaf produced with Rano Solina flour exhibited good dough development, a regular fermentation pattern, and a fine and homogeneous crumb structure (Figure 1).

The fermentation tests carried out on Rano Solina doughs enabled the monitoring of the weight, volume, height, and pH over time (Table 3). After 3 and 4 h, the final dough volume reached approximately 80–90 cm^3^, showing a moderate height increase and pH values close to the initial ones. These conditions resulted in products with limited acidity and an overall balanced sensory profile, although crumb development was not yet optimal. After 5 h, the dough reached its maximum volumetric expansion (>110 cm^3^), with a more than 1.5 cm height increase; however, this was accompanied by a more pronounced pH decrease (down to 5.2), indicative of intensified fermentation activity and perceptible acidity. After 6 h, the volume exceeded 140 cm^3^, but the substantial drop in pH (<5) corresponded to excessive acid development and sensory degradation.

The combined analysis of the data indicates that the optimal fermentation time for Rano Solina flour is between 3.5 and 4 h, representing a technological balance between dough expansion and acidification kinetics. Longer fermentation times resulted in a marked pH reduction, which may negatively affect dough stability and processing performance.

### 3.4. Prototype B—Pan Bauletto Autonomia B (Pre-Gelatinized Dough) Trials

**Figure 2:**
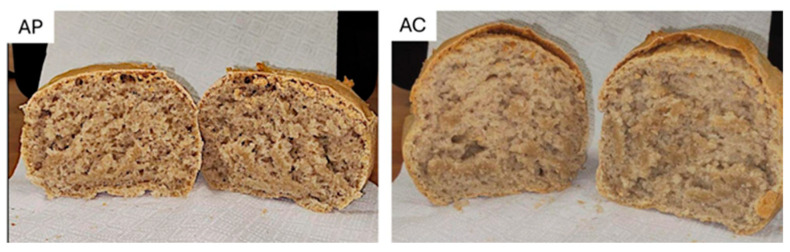
Cross-sectional views of bread samples produced with flour from the Autonomia B wheat cultivar: AP—bread obtained with pre-gelatinization of the dough; AC—bread obtained without pre-gelatinization, using a traditional process with 5 h of leavening.

Trials carried out with Autonomia B flour confirmed the technological limitations previously identified in the rheological characterization (i.e., low protein content and reduced gluten), indicative of a dough with low extensibility and a poor gas-holding capacity (Figure 2). Without pre-gelatinization, the doughs were compact, exhibited limited fermentation development and small, irregular alveolation, and produced loaves with pronounced acidity.

The incorporation of a pre-gelatinized fraction (10% of total flour) led to marked technological improvements. Both doughs showed progressive increases in height and volume (Table 4), but differences became evident at 5 h of fermentation. The standard dough reached a final volume of 221.20 cm^3^ (+139%), whereas the pre-gelatinized dough reached 203.62 cm^3^ (+120%). Although the maximum volumetric expansion was slightly lower in the pre-gelatinized dough, the crumb structure was more regular and the overall texture softer.

The pH decreased similarly in both doughs (from ~5.3 to 4.1–4.2 at 5 h), although the pre-gelatinized dough displayed slightly slower acidification, contributing to improved control of the perceived acidity.

#### Bread Sensory Profile

The present study also investigated the sensory profiles of breads produced with flour from the Autonomia B wheat cultivar, comparing a traditional process with 5 h of leavening to a variant incorporating a pre-gelatinization step (AC and AP, respectively). The evaluation was conducted using a descriptive approach with a trained sensory panel. For this purpose, a product characterization test was selected as capable of identifying which descriptors carry the most significant weight for judges in discriminating between products, based on quantitative intensity ratings. For each descriptor, an ANOVA model was fitted to test whether the judges’ scores differed significantly. The model assessed both the “product” effect and the “judge” effect for each measurement. Data processing generated coefficients for the selected model for each product–descriptor combination. In Table 5, the t-test values highlight the discriminating power of each descriptor in decreasing order, together with their corresponding *p*-values. The descriptors selected based on their discriminating ability were crust thickness, crumb color, alveolation, regularity of alveolation, loaf development, aroma intensity, acidity, crumb moisture, crumb elasticity, crumb gumminess, and crumb solubility. The *p*-values in the table highlight the attributes that contributed most effectively to discriminating (*p* < 0.05) between the two types of bread. Examining the values reported in Table 5, it is evident that the crust thickness, crumb color, and flavor intensity did not differ significantly between the two product types. In contrast, the kinesthetic attributes, together with acidity, exhibited marked discriminating power.

Table 6 shows the mean values and model coefficients for each combination of bread samples and descriptors. Each value relative to each descriptor indicates a significant discriminating effect—positive (blue) or negative (red)—regarding product characterization compared to the others analyzed. The colorless cells indicate descriptors that do not significantly characterize the product compared to the others. The results clearly indicate that the pre-gelatinization phase primarily affects the bread’s physical structure, yielding a more open, spongy crumb. This treatment improved the air pockets, enhancing both the elasticity and regularity.

The starch gel formed during heat treatment binds free water and releases it gradually during mixing and baking, thereby promoting a more stable and hydrated dough matrix. As confirmed during the sensory evaluation, pre-gelatinization contributes to greater softness and elasticity, with increased hydration visibly improving the crumb structure. Although this flour exhibits a high gluten index, it contains a very small total amount of gluten, which further complicates dough handling and development. This structural limitation makes the pre-gelatinization step particularly valuable, as it effectively compensates for the weak gluten network and markedly improves the rheological properties of the resulting bread. This technological effect is especially relevant for weak flours with low gluten content, where pentosans and pre-gelatinized starch help to create a compensatory structural network. At the molecular level, the pre-gelatinization process disrupts the native organization of starch granules, increasing their swelling capacity, solubility, and water-holding ability. These modifications optimize starch functionality, resulting in smoother and more stable gels that reduce hardness and enhance the elasticity in the final product. Moreover, pre-gelatinization significantly limits starch retrogradation, thereby slowing staling and improving moisture retention during storage. Overall, the technique enhances dough performance and contributes to the production of breads with superior textures, softness, and structural integrity.

The recent literature further supports these findings. A study [42] demonstrated that the pre-gelatinization of wholewheat flour induced by thermal fluidization significantly strengthened the gluten and starch networks in noodles, improving both cooking performance and textural attributes. In a subsequent investigation, the same authors [43] systematically evaluated the effects of pre-gelatinization on cooking behavior, texture, and digestibility, focusing on molecular changes in starch and proteins. Their comparative analysis revealed that thermal fluidization pre-gelatinization promoted the formation of dense gel networks and V-type crystalline complexes. Additionally, the gluten network exhibited increased structural strength, partly due to higher content of disulfide (S–S) bonds, enabling the treated products to achieve superior structural properties compared to untreated controls, despite having the same flour composition. The findings highlight that differences in processing performance, as evidenced by rheological parameters (Table 2), can significantly influence end-product quality and that targeted processing interventions may effectively compensate for less favorable technological characteristics.

### 3.5. Pasta Quality Assessment

#### 3.5.1. Dry Pasta Characterization

**Figure 3:**
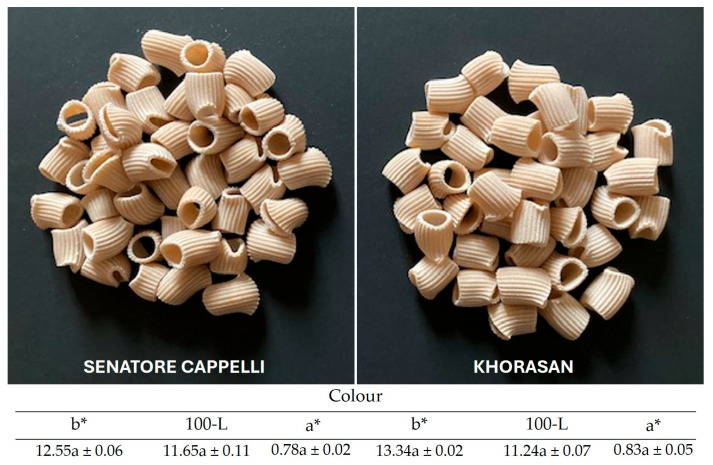
Dry pasta samples from *semolato* of durum wheat cv. Senatore Cappelli and Khorasan wheat. Color indices: yellow (b*), brown (100-L), and red (a*) ± standard deviation for ten replications. Means with different letters are significantly different (*p* ≤ 0.05) based on Duncan’s test.

Pasta samples from cvs Senatore Cappelli and Khorasan and their respective color indices are shown in Figure 3. The surfaces of both pasta samples appear to be red and opaque due to bronze die extrusion and the presence of bran. As mentioned before, the brightness (L*) and yellowness (b*) of pasta are important quality parameters for semolina pasta processors and consumers, and, in fact, the most suitable commercial durum wheat varieties to obtain high-quality pasta are characterized by b* values ≥ 20 [44]. In this study, despite the differences in color indices detected in the *semolato* from Senatore Cappelli and Khorasan wheat (Table 1), no significant differences were observed in the corresponding pasta samples (Figure 3). The artisanal manufacturing process adopted in this study likely did not allow the effective inhibition of the enzymes responsible for pigment degradation, mainly due to the absence of a vacuum mixing chamber in the plant. Indeed, during pasta processing, oxygen exposure, combined with relatively mild thermal conditions, may promote the activity of oxidative enzymes, particularly lipoxygenase (LOX) and peroxidase (POD), which catalyze the oxidation of carotenoid pigments naturally present in durum wheat semolina [45,46]. These oxidative reactions are recognized as one of the main causes of color loss during pasta-making. Moreover, high-temperature drying conditions or the use of alternative drying strategies, such as vacuum drying or multi-stage temperature ramping, have been shown to further inactivate oxidative enzymes, contributing to color retention and higher b* values in the final pasta product, as well as to improved firmness, higher water uptake, and lower cooking loss [47,48]. In contrast, the very low drying temperatures used in this study (42 °C) likely enhanced enzymatic activity, accelerating pigment degradation and resulting in the reduced yellowness of the pasta. Indeed, a decrease in the b* value of 35 and 26% from the *semolato* to pasta was observed in Khorasan and Senatore Cappelli, respectively.

#### 3.5.2. Pasta Cooking Quality

**Figure 4:**
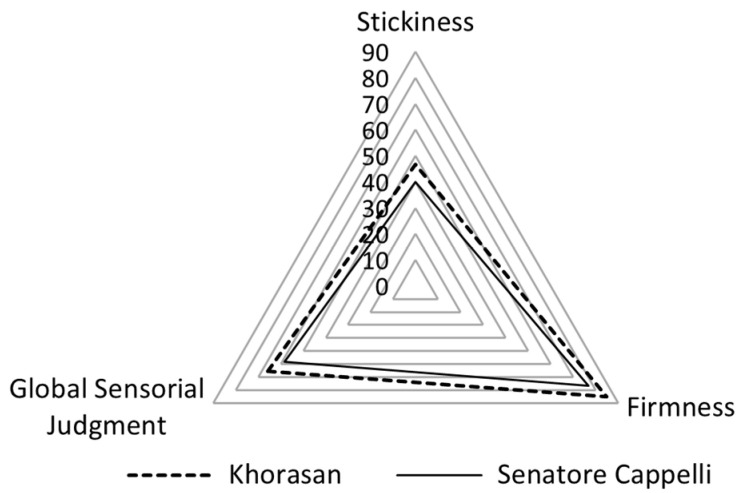
Radar chart of sensory assessment of cooked pasta from durum wheat cv. Senatore Cappelli and Khorasan wheat.

The optimal cooking time for both pasta samples was 13 min. Pasta from Senatore Cappelli showed a higher value for water absorption (74 g/100 g) compared to Khorasan wheat (68 g/100 g). Generally, the weight of semolina cooked pasta was 2.4–2.6 times that of the dry semolina pasta, corresponding to total water absorption of approximately 140–160% of its original weight [48]. The values detected in this study were significantly lower, probably due to the higher presence of bran in the *semolato* compared to semolina. Indeed, previous observations [49,50] indicated that wholemeal pasta generally showed lower water absorption when compared to its semolina counterpart, since bran competes with starch for water. Moreover, the low drying temperature applied during pasta production (T max = 42 °C) may partly explain the reduced water absorption capacity of the pasta samples, as increasing drying temperatures are reported to promote higher water uptake in cooked pasta [51,52]. The sensory attributes of the cooked pasta from cv. Senatore Cappelli and Khorasan wheat are reported in Figure 4. Firmness and stickiness represent the most important quality attributes for Italian consumers, requiring pasta to be firm to the bite, *al dente*, and not sticky during mastication. In contrast, such characteristics are not necessarily prioritized by international consumers, who may instead prefer a less resistant texture, consistent with the culinary habits and cooking practices of their own countries. The sensory analysis highlighted differences between the two pasta samples. The Khorasan pasta received “very good” scores for firmness (85), associated with low stickiness (47), whereas the pasta from Senatore Cappelli exhibited reduced firmness (77) and stickiness (40). The Khorasan pasta received higher scores for overall acceptability (GSJ = 66) compared to Senatore Cappelli (GSJ = 58).

## 4. Conclusions

Taken together, the present results clearly demonstrate the good technological performance of ancient wheat varieties. Beyond this, the results support the broader role of ancient wheat varieties in reinforcing local food micro-economies, promoting biodiversity conservation, and contributing to sustainable dietary patterns. By integrating appropriate processing strategies with short supply chains and artisanal production, ancient wheats can be successfully repositioned as high-added-value products, strengthening their roles within agroecological and climate-resilient food systems. Overall, the results of this study indicate that Italian ancient wheat varieties represent viable raw materials for the production of high-quality wholemeal breads and pasta within organic and low-input agri-food systems. The findings confirm the distinctive attributes of ancient wheat varieties, characterized by high protein content even under organic cultivation systems. However, their low gluten indices limit their suitability for extended fermentation processes. Nevertheless, it should be taken into account that the term “weak gluten” should not be misconstrued as indicating poor quality; rather, it denotes suitability for specific processing sectors, such as pasta, flatbreads, biscuits, and certain pastry products. Moreover, consumers increasingly perceive ancient grains as traditional, local, and sustainable and are willing to pay a premium for such attributes. To address the technological limitations, several evidence-based strategies can be employed, ranging from blending strategies and the optimization of process parameters to selection and breeding among ancient wheat germplasms. In conclusion, while weaker gluten strength represents the primary technological limitation of ancient wheat varieties, multiple scientifically validated strategies [20] are available to mitigate these constraints. When appropriately applied, these approaches will allow the development of bread and pasta products with satisfactory technological performance, while preserving the nutritional, sensory, and sustainability-related advantages that make ancient wheats attractive in contemporary markets. Future research should be aimed at scientifically substantiating the premium positioning of ancient wheat-based foods through a multidisciplinary approach that might integrate agronomic systems, nutritional profiling, technological processes, and measurable health-related claims.

## Figures and Tables

**Table 1:** Ash and protein content and color indices of flour and *semolato* from varieties of bread, durum and Khorasan wheat.

	Variety	Ash (g/100 g d.m.)	Proteins (g/100 g d.m.)	Colour		
				b*	100-L	a*
**FLOUR**	Andriolo	0.865 ± 0.007 ^c^	11.535 ± 0.007 ^c^	10.71 ± 0.06 ^a^	7.94 ± 0.05 ^c^	−0.25 ± 0.03 ^c^
	Autonomia B	0.73 ± 0.00 ^d^	10.725 ± 0.007 ^d^	6.02 ± 0.08 ^c^	6.23 ± 0.22 ^d^	0.16 ± 0.01 ^b^
	Gentil Rosso	1.00 ± 0.00 ^b^	12.61 ± 0.014 ^b^	10.29 ± 0.09 ^a^	9.49 ± 0.10 ^a^	0.31 ± 0.06 ^a^
	Maiorca	1.125 ± 0.007 ^a^	12.91 ± 0.014 ^b^	10.50 ± 0.26 ^a^	8.02 ± 0.06 ^b^	−0.27 ± 0.04 ^c^
	Rano Solina	0.86 ± 0.00 ^c^	15.90 ± 0.20 ^a^	9.02 ± 0.07 ^b^	8.04 ± 0.08 ^b^	0.09 ± 0.06 ^b^
	Verna	0.855 ± 0.007 ^c^	11.395 ± 0.007 ^c^	10.91 ± 0.16 ^a^	8.01 ± 0.27 ^b^	−0.23 ± 0.11 ^c^
***SEMOLATO***	Khorasan	1.345 ± 0.007 ^C^	10.45 ± 0.00 ^C^	20.57 ± 0.16 ^A^	12.8 ± 0.25 ^B^	0.14 ± 0.09 ^C^
	Senatore Cappelli	1.57 ± 0.00 ^A^	12.90 ± 0.03 ^A^	16.92 ± 0.20 ^B^	13.49 ± 0.06 ^A^	0.43 ± 0.0 2 ^B^
	Timilia	1.50 ± 0.014 ^AB^	11.04 ± 0.03 ^B^	13.14 ± 0.20 ^C^	12.99 ± 0.26 ^B^	1.30 ± 0.09 ^A^

Results are expressed as mean ± standard deviation for three replications. Within the same column, means with different letters (lowercase for common wheat flour and uppercase for durum wheat *semolato*) are significantly different (*p* ≤ 0.05) based on Duncan’s test.

**Table 2:** SDS sedimentation test findings, gluten index (GI) values, alveographic and farinograph parameters, and falling numbers (FN) of flour and *semolato* from varieties of common, durum, and khorasan wheat.

	Variety	SDS (mL)	GI (%)	Alveograph			Farinograph					FN (s)
				W (J × 10^−4^)	P/L	Ie (%)	Water Absorption (%)	Development Time (min)	Stability (min)	Softening		
										At 10 min	At 12 min	
**FLOUR**	**Andriolo**	53.0 ± 0.0 ^a^	27 ± 1 ^c^	78 ± 8 ^b^	0.39 ± 0.06 ^c^	33.1 ± 1.5 ^a^	52.3 ± 0.4 ^c^	1.7 ± 0.1 ^c^	2.7 ± 0.1 ^a^	89.0 ± 0.6 ^d^	104.0 ± 0.8 ^d^	403 ± 2 ^b^
	**Autonomia B**	35.5 ± 0.7 ^e^	92 ± 6 ^a^	87 ± 4 ^a^	1.10 ± 0.03 ^a^	32.2 ± 0.5 ^a^	55.7 ± 0.1 ^b^	1.5 ± 0.0 ^c^	1.3 ± 0.6 ^d^	98.0 ± 0.8 ^c^	122.0 ± 1.4 ^b^	313 ± 6 ^f^
	**Gentil Rosso**	44.0 ± 0.0 ^c^	3 ± 0 ^e^	55 ± 2 ^d^	0.9 ± 0.1 ^a^	16.6 ± 0.4 ^c^	55.5 ± 0.7 ^b^	2.2 ± 0.3 ^b^	1.9 ± 0.1 ^c^	111.0 ± 2.5 ^b^	126.0 ± 2.3 ^b^	440 ± 4 ^a^
	**Maiorca**	41.0 ± 1.4 ^d^	4 ± 1 ^e^	47 ± 2 ^e^	1.1 ± 0.1 ^a^	0.0	58.5 ± 0.5 ^a^	2.2 ± 0.5 ^b^	1.7 ± 0.3 ^cd^	124.0 ± 0.7 ^a^	139.0 ± 0.8 ^a^	342 ± 8 ^d^
	**Rano Solina**	50.0 ± 0.0 ^b^	41.5 ± 2.1 ^b^	61 ± 3 ^c^	0.71 ± 0.07 ^b^	23.2 ± 1 ^b^	59.0 ± 0.1 ^a^	3.5 ± 0.4 ^a^	2.4 ± 0.3 ^ab^	90.0 ± 0.0 ^d^	113.0 ± 2.8 ^c^	330 ± 3 ^e^
	**Verna**	50.0 ± 0.0 ^b^	18 ± 1 ^d^	80 ± 5 ^ab^	0.45 ± 0.06 ^c^	31.3 ± 0.7 ^a^	53.1 ± 1.0 ^c^	2.0 ± 0.0 ^b^	2.3 ± 0.0 ^b^	107.0 ± 2 ^b^	117.0 ± 0.0 ^c^	374 ± 10 ^c^
***SEMOL***	**Khorasan**	22.0 ± 0.0 ^B^	5 ± 0 ^A^	57 ± 5 ^A^	1.6 ± 0.2 ^B^	0.0	55.8 ± 1.1 ^B^	2.0 ± 0.0 ^B^	3.0 ± 0.7 ^A^	76.0 ± 0.2 ^C^	82.0 ± 0.7 ^C^	591 ± 3 ^A^
	**Senatore Cappelli**	28.0 ± 0.0 ^A^	6 ± 0 ^A^	55 ± 1 ^A^	2.0 ± 0.1 ^A^	0.0	62.0 ± 0.0 ^A^	2.5 ± 0.6 ^A^	1.9 ± 0.1 ^C^	110.0 ± 0.0 ^A^	126.0 ± 0.0 ^A^	473 ± 5 ^C^
	**Timilia**	27.5 ± 0.7 ^A^	5.5 ± 2.1 ^A^	37 ± 2 ^B^	1.06 ± 0.12 ^C^	0.0	55.3 ± 0.8 ^B^	2.2 ± 0.6 ^AB^	2.5 ± 0.0 ^AB^	97 ± 3 ^B^	111.0 ± 0.0 ^B^	514 ± 8 ^B^

Results are expressed as mean ± standard deviation for three replications. Within the same column, means with different letters (lowercase for common wheat flour and uppercase for durum wheat *semolato*) are significantly different (*p* ≤ 0.05) based on Duncan’s test. Ie = elasticity index.

**Table 3:** Evolution of Rano Solina dough according to piece weight, height, volume, and pH during fermentation from 3 to 6 h. Percentage variations were calculated relative to the corresponding initial values measured at each time point (initial height, initial volume, initial pH).

Hours of Fermentation	Weight (g)	Initial Height (cm)	Final Height (cm)	Initial Volume (cm^3^)	Final Volume (cm^3^)	Initial pH	Final pH
**3 h**	52.43 ± 0.41 ^b^	1.30 ± 0.05 ^ab^	1.72 ± 0.08 ^d^	61.09 ± 1.25 ^a^	79.60 ± 2.40 ^d^	5.32 ± 0.04 ^ns^	4.69 ± 0.06 ^a^
Delta (%)		32.3		30.3		−11.8	
**4 h**	50.13 ± 0.36 ^b^	1.50 ± 0.06 ^a^	2.29 ± 0.10 ^c^	55.34 ± 1.10 ^b^	86.21 ± 2.85 ^c^	5.28 ± 0.05 ^ns^	4.27 ± 0.05 ^ab^
Delta (%)		52.7		55.7		−19.1	
**5 h**	50.51 ± 0.48 ^b^	1.20 ± 0.05 ^b^	2.50 ± 0.12 ^b^	55.53 ± 1.45 ^b^	115.69 ± 3.90 ^b^	5.35 ± 0.03 ^ns^	4.16 ± 0.04 ^bc^
Delta (%)		108.3		108.3		−22.2	
**6 h**	56.25 ± 0.52 ^a^	1.30 ± 0.04 ^ab^	3.20 ± 0.15 ^a^	59.23 ± 1.60 ^a^	148.09 ± 4.75 ^a^	5.36 ± 0.04 ^ns^	4.02 ± 0.05 ^c^
Delta (%)		164.2		149.9		−25	

Values are expressed as mean ± standard deviation of three independent replicates. Within each row, means followed by different letters are significantly different (*p* ≤ 0.05).

**Table 4:** Changes in height, volume, and pH of standard (AC) and pre-gelatinized (AP) Autonomia B dough pieces during fermentation (0–5 h).

Hours of Fermentation	Weight (g)	Height (cm)		Volume (cm^3^)		pH	
		AC	AP	AC	AP	AC	AP
**0 h**	100 ± 1.3	2.00 ± 0.06 ^cA^	2.00 ± 0.05 ^cA^	92.55 ± 1.85 ^dA^	92.55 ± 1.90 ^dA^	5.30 ± 0.04 ^aA^	5.27 ± 0.05 ^aA^
**3 h**	100 ± 1.5	2.80 ± 0.09 ^cA^	2.80 ± 0.08 ^cA^	129.58 ± 3.10 ^cA^	129.58 ± 2.95 ^cA^	4.68 ± 0.06 ^bA^	4.73 ± 0.05 ^bA^
Delta (%)		40	40	40	40	−11.7	−10.2
**4 h**	100 ± 1.9	3.90 ± 0.12 ^bA^	3.90 ± 0.11 ^bA^	180.48 ± 4.20 ^bA^	180.48 ± 4.05 ^bA^	4.44 ± 0.05 ^cA^	4.41 ± 0.04 ^cA^
Delta (%)		95	95	95.2	95.2	−16.2	−16.3
**5 h**	100 ± 1.3	4.78 ± 0.16 ^aA^	4.40 ± 0.14 ^aB^	221.20 ± 5.60 ^aA^	203.62 ± 5.10 ^aB^	4.10 ± 0.05 ^dB^	4.20 ± 0.06 ^cA^
Delta (%)		139	120	139.1	120	−22.6	−20.03

Delta values are expressed as percentage variation relative to the initial time (0 h). Values are expressed as mean ± standard deviation of three independent replicates. Different lowercase letters indicate significant differences among fermentation times within the same dough type, whereas different uppercase letters indicate significant differences between dough types at the same fermentation time (*p* ≤ 0.05).

**Table 5:** Discriminating power of sensory descriptors obtained from the descriptive analysis of Autonomia B breads (traditional process vs. pre-gelatinized dough).

Descriptor	Test Value	*p*-Value
Homogeneity	5.645	0.000
Development	5.459	0.000
Alveoli regularity	5.388	0.000
Acidity	4.538	0.000
Crumb moisture	4.420	0.000
Crumb solubility	4.215	0.000
Crumb elasticity	3.793	0.000
Alveoli	3.545	0.000
Crust thickness	−0.003	0.501
Crumb color	−0.528	0.701
Flavor intensity	−0.818	0.793

Descriptors are listed in decreasing order of discriminating power. Significant *p*-values (*p* ≤ 0.05) indicate descriptors contributing significantly to product differentiation.

**Table 6:** Adjusted mean values of sensory descriptors for the bread obtained with pre-gelatinization (AP) and without pre-gelatinization (AC). Cell colors indicate the positive (blue) or negative (red) association of the descriptor with the sensory profile of the product.

Descriptor	AP	AC
Crumb color	2.67	2.57
Homogeneity	3.67	2.21
Alveoli	3.16	2.18
Alveoli regularity	3.11	2.03
Development	4.04	2.10
Crumb moisture	4.17	3.17
Crumb elasticity	3.50	2.54
Flavor intensity	3.03	3.07
Crumb solubility	2.25	3.21
Crust thickness	2.33	2.53
Acidity	1.92	3.05

## Data Availability

The original contributions presented in this study are included in the article. Further inquiries can be directed to the corresponding author.
